# 

**Published:** 1877-11

**Authors:** 


### CONTENTS.

### COMMUNICATIONS.

On the Formation of Dentine........ 449
Connection between Excessive Brain worry and Bodily Disease .. .. .. 454
Professional Status and Education........ 459
Introductory Lecture. Ohio College of Dental Surgery........ 462
Air Chambers........ 468
Injurious Effects of Vulcanized Rubber........ 470
Hints for Working Celluloid........ 471

### SELECTIONS.

Grafts of the Dental Follicles........ 473
Force and its Transmutations........ 477
The Germ Doctrine and Septicæmia........ 480
Properties of the Human Gastric Juice........ 481
Experiments with the Turkish Bath........ 482
Nerve Stretching in Neuralgia........ 483
Removing Foreign Bodies from the Æsophagus........ 484
Diameter of the Red Blood Corpuscles........ 484
How to Deprive Iodine of its Stain........ 485
Sewer Gases........ 486
Paris Green Poisoning Case........ 486
Disinfection by Heat........ 487
Hypodermic Injection of Ammonia for Opium Poisoning........ 478
Vinegar in Opium Poisoning........ 487

### EDITORIAL.

Correction........ 489
Examiners' Meeting........ 489
Nomenclature........ 489
Ohio Dental Society........ 490
Good-Bye—The Editor Retires........ 491

